# Erratum

**DOI:** 10.1111/jdi.13109

**Published:** 2019-09-01

The authors would like to draw the reader's attention to the errors in the following article.

Kijmanawat A, Panburana P, Reutrakul S, Tangshewinsirikul C. Effects of probiotic supplements on insulin resistance in gestational diabetes mellitus: A double‐blind randomized controlled trial. *J Diabetes Investig* 2019; 10: 163–170.

On pages 163 (under ‘Materials and Methods’) and 164 (under ‘METHODS’), the period that a double‐blind, placebo‐controlled, randomized clinical trial was carried out should have been between July 2016 and February 2017.

In Figure 1 on page 166, the period that GDM patients at GA 24 – 28 weeks were in Ramathibodi Hospital should have been between July 2016 and February 2017.

The authors apologize for these errors and any confusion they may have caused.

